# Elongation of Müllerian ducts and connection to urogenital sinus determine the borderline of uterine and vaginal development

**DOI:** 10.1016/j.bbrep.2018.10.013

**Published:** 2018-11-30

**Authors:** Tadaaki Nakajima, Risa Yamanaka, Yasuhiro Tomooka

**Affiliations:** a Department of Biological Science and Technology, Faculty of Industrial Science and Technology, Tokyo University of Science, 6-3-1 Niijuku, Katsushika-ku, Tokyo 125-8585, Japan; b Institute of Industrial Science, The University of Tokyo, 4-6-1 Komaba, Meguro-ku, Tokyo 153-8505, Japan

**Keywords:** Uterus, Vagina, Müllerian duct, Retinoic acid, Morphogenesis

## Abstract

In female mice, proximal, middle and caudal Müllerian ducts (MDs) differentiate into oviduct, uterus and vagina, respectively. The fates of female reproductive tract epithelia are determined by the mesenchyme. However, the mesenchymal fate determination system is still unclear. It is reported that presence or absence of retinoic acid (RA) signaling in MD mesenchyme induced uterine or vaginal mesenchyme, respectively. To analyze determination of the borderline, RA signal switching factors were found to play critical roles. Expression of a RA metabolizing enzyme, CYP26A1, was high in the epithelium of caudal MD and urogenital sinus, indicating that the enzyme causes the absence of RA signaling in the region. mRNA expression of some transcription factors regulating *Aldh1a2*, RA synthesis enzyme expressed in MDs, in other tissues was detected in MDs. When the transcription factor genes were overexpressed in a uterine mesenchymal cell line, *C/ebpδ* overexpression stimulated *Aldh1a2* expression. Furthermore, C/EBPδ protein was strongly expressed in the proximal and middle regions of the MDs and bound to the *Aldh1a2* promoter in vivo. Since *C/ebpδ* mRNA expression was maintained at the same level in proximal, middle and caudal MDs, we hypothesize that a high frequency of mitosis induces a low level protein expression in MD mesenchyme. In fact, the mitotic activity was significantly high in caudal mesenchyme, and a mathematical model showed that a gradient of protein was induced by cell proliferation. Therefore, morphogenesis of MDs controls the fate of mesenchyme via RA degradation in urogenital sinus and a gradient of proteins involved in RA synthesis.

## Highlights

• Degradation of RA by CYP26A1 is involved in fate determination of vaginal mesenchyme.
• C/EBPδ can directly bind to *Aldh1a2* promoter and stimulates the expression.
• Müllerian ductal cell proliferation causes protein gradient involved in RA synthesis.

## Graphical abstract

**Figure:**
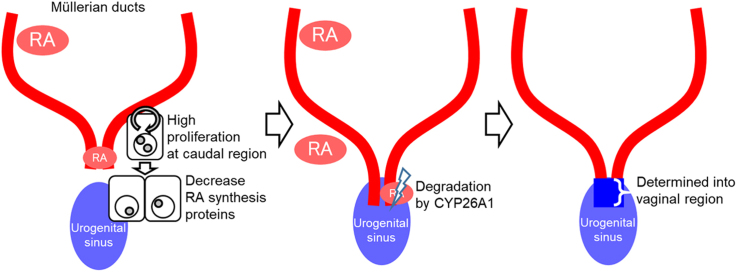

## 1 Introduction

In female mice, Müllerian ducts (MDs) initiate growth at the proximal region of *meso*nephros derived from mesoderm and elongate into urogenital sinus (UGS) derived from endoderm [1]. The proximal MD region of is adjacent to gonads and differentiates into oviducts, and the middle region distinguished from other tissues differentiates into uterus [2]. Although vaginal epithelium is derived from MD epithelium, vagina develops from caudal MD which are combined with UGS [3]. These female reproductive organs have their own morphology and function. Oviductal epithelium consists of ciliated cells and secretory cells [4], and uterine epithelium is composed of simple columnar luminal and glandular epithelial cells and vaginal epithelium develops into stratified cuboidal epithelium with cornification. Hetero-recombinations of epithelium and mesenchyme between uterus and vagina demonstrated that the fate of MD epithelial cells was determined by the mesenchyme up to postnatal day 7 (P7) [5], [6]. However, the mechanism of the fate determination of the mesenchyme themselves had been unclear.

Homeobox (*Hox*) cluster genes are most analyzed factors for development of MD mesenchyme. *Hoxa9* is expressed at high levels in regions that will become oviducts, *Hoxa10* is expressed in the development of uterus, *Hoxa11* is expressed in the primordial lower uterus and cervix, and *Hoxa13* is expressed in the cervix and upper vagina [7]. *Hoxa10* deficiency induces an oviduct-like structure only in anterior part of uterus [8], and *Hoxa13* deficiency causes hypoplastic urogenital genital sinus and agenesis of posterior part of MD, but not affects the differentiation [9]. The *Hox* genes are also involved in adult functions of female reproductive tracts (e.g. implantation) [7]. These reports suggest that the *Hox* genes are mainly responsible for adult function in female reproductive tract, but they are not main factors that determined regional fate of MD mesenchyme.

Retinoic acid (RA) is an essential component of cell-cell signaling during organogenesis [10]. Vitamin A deficient mice and RA receptor/retinoid X receptor mutant mice exhibits a complete absence of MDs, indicating that RA is essential for development of MDs [11], [12], [13]. RA is synthesized from retinol in an oxidation process catalyzed by alcohol dehydrogenases and aldehyde dehydrogenases [14], [15]. RA is metabolized to hydroxylated forms by cytochrome P450, family 26, subfamily a, polypeptide 1 (CYP26A1) and CYP26B1 [16], [17], and the metabolism of RA attenuated the activity of binding to RA receptors [18]. In MDs, expressions of retinol dehydrogenase 10 (RDH10), aldehyde dehydrogenase family 1, subfamily A2 (ALDH1A2) and RA signaling in the proximal and middle mesenchyme are higher than those in the caudal mesenchyme [2]. Furthermore, presence or absence of RA signaling is the fate-determining factor of MD mesenchyme into uterine or vaginal mesenchyme, respectively [2]. However, it is unclear why RA signaling disappears from the caudal MD mesenchyme and what induces RA synthesis enzymes in development of the mesenchyme.

In the present study, regulation mechanisms of RA metabolism and synthesis were analyzed in MDs. The expression profile of CYP26 in MDs was investigated, and candidates of transcription factors that could be upstream of *Rdh10* or *Aldh1a2* in MDs were searched. Roles of the candidates in *Rdh10* and *Aldh1a2* expression are then demonstrated.

## 2 Materials and methods

### 2.1 Animals

Female CD-1 mice (Sankyo Lab, Tokyo, Japan) were given a commercial diet and tap water *ad labium* and kept at 22–24°C under 12 h light/12 h darkness by artificial illumination (lights on 08:00–20:00). Animals were maintained in accordance with the NIH Guide for the Care and Use of Laboratory Animals and were approved by our institutional Animal Care Committee. The presence of vaginal plugs indicated embryonic day 0.5 (E0.5). MDs at E12.5 were not selected for male or female. Spleens were dissected from adult mice.

### 2.2 Cell culture

P3US cells were established from uterine mesenchyme of *p53*^*-/-*^ mice at P3 and cultured in 1:1 mixture of Dulbecco modified Eagle medium and Ham nutrient mixture F-12 without phenol red (Sigma-Aldrich, St. Louis, MO, USA) containing heat-inactivated FBS (Cell Culture Technologies, Zurich, Switzerland) at 10%, penicillin (31 μg/ml, Sigma-Aldrich) and streptomycin (50 μg/ml, Sigma-Aldrich) (10% FBS medium) in a humidified atmosphere of 5% CO_2_ at 37°C. The cells were passaged using 0.05% trypsin-0.02% EDTA (Sigma-Aldrich).

### 2.3 RNA isolation and reverse transcript (RT)-PCR

**Fig. 1:**
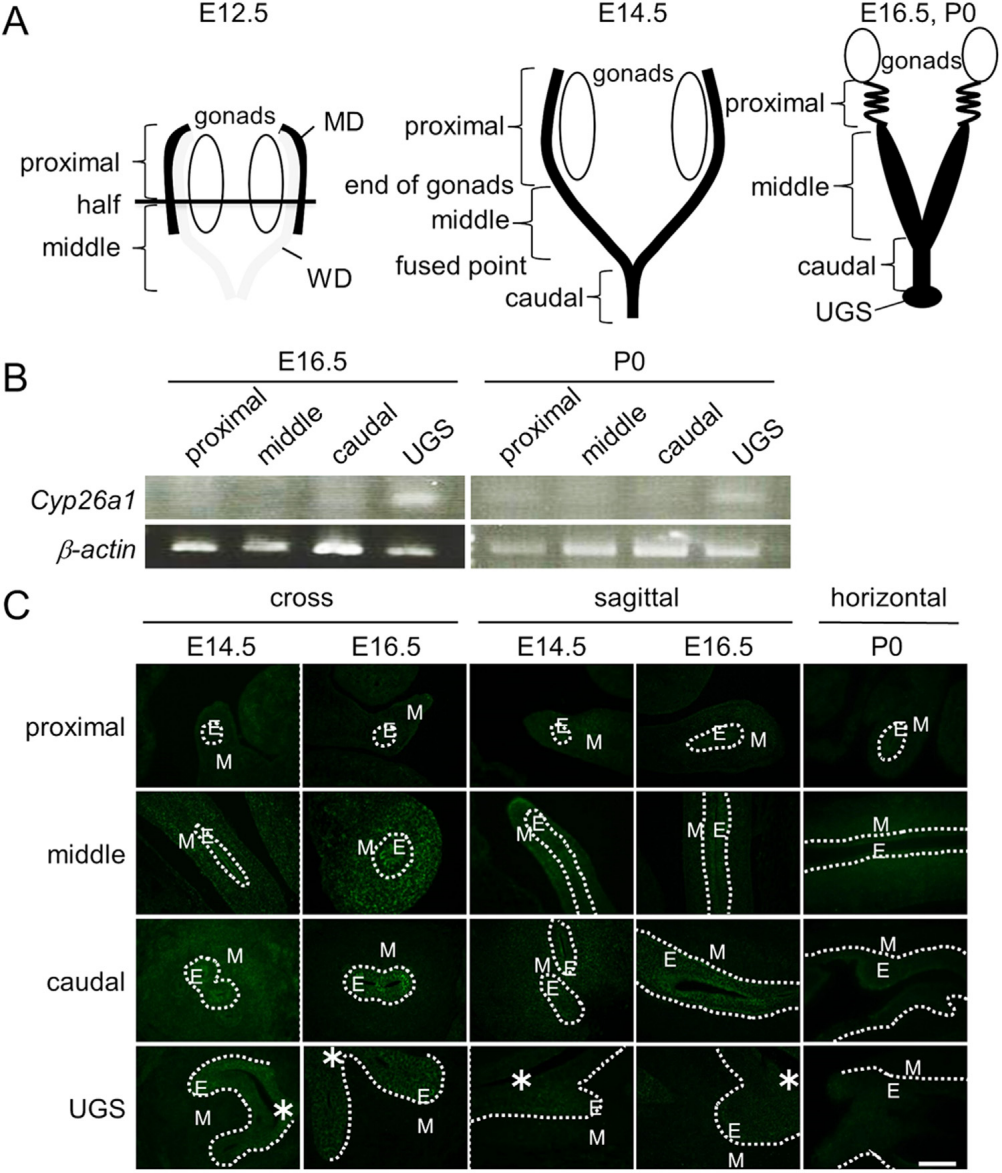
Isolation of Müllerian ducts for RT-PCR. (A) Ontogenic expression of *Cyp26a1* mRNA in Müllerian ducts and UGS by RT-PCR. (B) Ontogenic expression of CYP26A1 protein in Müllerian ducts and UGS at E14.5, E16.5 and P0 by immunohistochemistry. (C) Green: CYP26A1 positive cells. White line: borderline between the epithelium and mesenchyme. E: epithelium. M: mesenchyme. *: urethral tube. Scale bar: 100 µm. (n = 3).

Total RNA was isolated from MDs and UGS at E12.5 and P0 (Fig. 1A) or P3US cells by acid guanidinium-phenol-chloroform extraction. RT was performed with ReverTra Ace qPCR RT Master Mix with gDNA Remover (TOYOBO, Osaka, Japan). PCR was carried out with Amplitaq Gold PCR Master Mix (Applied Biosystems, Foster City, CA, USA) and specific primers (Supplemental table). *β-actin* was chosen as an internal standard. Ten-thirteen MDs or UGS were pooled in one sample.

### 2.4 Overexpression of transcription factors in P3US cells

RNA isolation and RT were performed from the middle of MDs at E16.5 as described above. ORFs were amplified with TksGflex DNA Polymerase (TaKaRa, Shiga, Japan) with specific primers (Supplemental table), and inserted into pcDNA 3.1 Hygro (+) plasmids using Ligation high ver.2 (TOYOBO). Each gene was sequenced using the BigDye Terminator v3.1 Cycle Sequencing kit and analyzed with 3130xl Genetic Analyzer (Applied Biosystems).

P3US cells in 60 mm dishes were transiently transfected with 1 μg of these plasmids by Screen Fect A (Wako, Osaka, Japan) according to the manufacturer's instructions. Control cells were P3US cells transfected with pcDNA 3.1 Hygro (+) vector. After 48 h, gene expressions in these cell lines were analyzed by RT-PCR.

### 2.5 ChIP assay

Ten MDs at E14.5 were sampled in 1% Protease Inhibitor Cooktail (Wako; added to all buffer by chromatin extraction)/PBS, and crosslinked with 1.5% PFA. Fixation was stopped by addition of glycine. The samples were washed with PBS and homogenized. The pellets were lysed in 0.5% NP40 buffer (10 mM Tris (pH8.0), 10 mM NaCl), and then lysed in 1% SDS buffer (50 mM Tris (pH8.0), 10 mM EDTA). The samples were added with ChIP dilution buffer (50 mM Tris (pH8.0), 167 mM NaCl, 1.1% Triton X-100 and 0.11% Sodium Deoxycholate). The chromatins were sheared with a sonicator to an average length of 200 bp.

Thirty μl ChIP-grade protein G magnetic beads (Cell Signaling Technologies, Beverly, MA, USA) and 0.5 μg anti-RNA polymerase II CTD repeat antibody (Santa Cruz biotechnology, Santa cruz, CA, USA), anti-CEBPδ antibody (Santa Cruz Biotechnology) or normal rabbit IgG (Santa Cruz Biotechnology) were mixed in RIPA buffer and incubated at 4°C overnight. After washing with RIPA buffer, the mixtures and the fragmented chromatins were mixed and incubated at 4°C overnight. Samples were then sequentially washed in RIPA buffer, RIPA buffer containing 500 mM NaCl, LiCl wash solution and TE buffer, and eluted in 0.5% SDS buffer (10 mM Tris (pH 8.0), 300 mM NaCl, 5 mM EDTA) at 65°C for 8 h, then added with protease K at 500 μg/ml and incubated for 1 h at 55°C. Proteins were removed by phenol and chloroform and DNAs were precipitated by ethanol. The DNAs were compared with inputs by quantitative PCR using KOD SYBR qPCR Mix (TOYOBO). *Aldh1a2* TSS (−89 to +11), *Aldh1a2* C/EBPδ predicted binding site (+2872 to +3987) and *Aldh1a2* conserved region primer (−381 to +1, 25,794 to +26,539) were derived from Gene ID 19,378 (Supplemental Table).

### 2.6 Histology

To detect CYP26A1, female mice at E14.5, E16.5 and P0 were fixed in 4% PFA at 4°C for 3 h. The samples were embedded in paraffin and cut into 6 µm sections. To detect C/EBPδ, female mice at E14.5 and spleens for positive control were embedded in O.C.T. Compound and frozen by liquid nitrogen. The cryosections (10 µm) were fixed in 4% PFA at room temperature for 5 min. To detect CYP26A1, the sections were microwaved in 10 mM sodium citrate buffer (pH 6.0) for 10 min. Nonspecific binding was blocked in PBS containing 5% goat serum and 1% BSA for 30 min. The sections were incubated at 4°C overnight with primary antibody for CYP26A1 (1/200; ALPHA DIAGNOSTIC, San Antonio, TX, USA) in PBS containing 0.1% Triton X-100, 1% BSA and 0.1% nonfat dried milk, C/EBPδ (1/50; Abcam, Cambridge, UK) in PBS containing 5% goat serum and 1% BSA, and then incubated with Alexa488-conjugated anti-rabbit IgG goat antibody (1/250; Jackson Immuno Research, West Grove, PA, USA). For negative control, normal rabbit IgG (Santa Cruz Blotechnology) was used. DAPI was used to stain nucleic acids.

To detect cell proliferation, pregnant mice at day of post coitus 14.5 were injected with 100 μg/0.1 ml/20 gBW EdU (Thermo Fisher, Waltham, MA, USA). After administration for 1 h, female embryos were fixed in 4% PFA at 4°C overnight (n = 5–7). The samples were embedded in paraffin and cut into 6 µm sections. The sections were stained with Click-iT EdU Alexa Fluor 594 Imaging Kit (Thermo Fisher), according to manufacturer’s instructions. The number of EdU-positive cells and Hoechst-stained cells were counted manually. All of epithelial cells in the 1–3 pictures and 200 mesenchymal cells were counted in a section.

### 2.7 Mathematical modeling

A one dimensional cell-based MD ductal elongation model was generated. In this model, cells line one dimensionally and cell proliferation occurs from proximal to caudal region. (Fig. 4C). First, 10 cells have constant levels of mRNA or protein (the level indicated as “**C**”), and frequency of cell proliferation is set as 18, 24 or 30% in proximal (2/5 head region in all cells at the time), middle (2/5 following region) or caudal region (1/5 last region). mRNA or protein degradation levels are indicated as “**d**”, and the synthesis levels are indicated as “**s**”, and **d** and **s** are hypothesized at constant.

$$C_{n}=\{\begin{array}{cc} (1-d)C_{n-1}+s, & \left(not\;proliferated\right)\\ \frac{(1-d)C_{n-1}}{2}+s, & \left(proliferated\right) \end{array}$$

When the number of cells reaches more than 5000, **C** is averaged each in 100 cells and shows as dots graphically (n = 10).

### 2.8 Statistical analysis

Data were expressed as mean ± standard error. Two-tailed Student’s *t*-test or Welch’s *t*-test was used for single comparisons. For multiple comparisons, differences were estimated using ANOVA with appropriate *post hoc* tests. A statistically significant difference was defined as *p* ≤ 0.05.

## 3 Results

### 3.1 Ontogenic expression of CYP26 mRNA and protein in MDs

The expression of RA metabolizing enzyme gene, *Cyp26*, was investigated in proximal, middle and caudal MDs and UGS at E16.5 and P0 (Fig. 1B). *Cyp26a1* was mainly expressed in UGS and slightly expressed in proximal and caudal MD. *Cyp26b1* was not detected in MDs and UGS at E16.5 and P0 (data not shown).

CYP26A1 protein expression was investigated in cross, sagittal or horizontal sections of MDs and UGS at E14.5, E16.5 and P0 (Fig. 1C). CYP26A1 was expressed in cytoplasm and not expressed in nuclei at all stages. CYP26A1 was mainly expressed in mesenchyme of MDs. In caudal MD and UGS, the enzyme expression was strongly detected in the epithelium at embryonic stages. The expression pattern of CYP26A1 was not changed at P0, but the expression levels were decreased. Therefore, RA metabolizing enzyme gene and protein were highly expressed at caudal MD and UGS at embryonic stages.

### 3.2 Analysis of ontogenic expression of transcription factors in upstream of *Aldh1a2* mRNA in MDs by RT-PCR

**Fig. 2:**
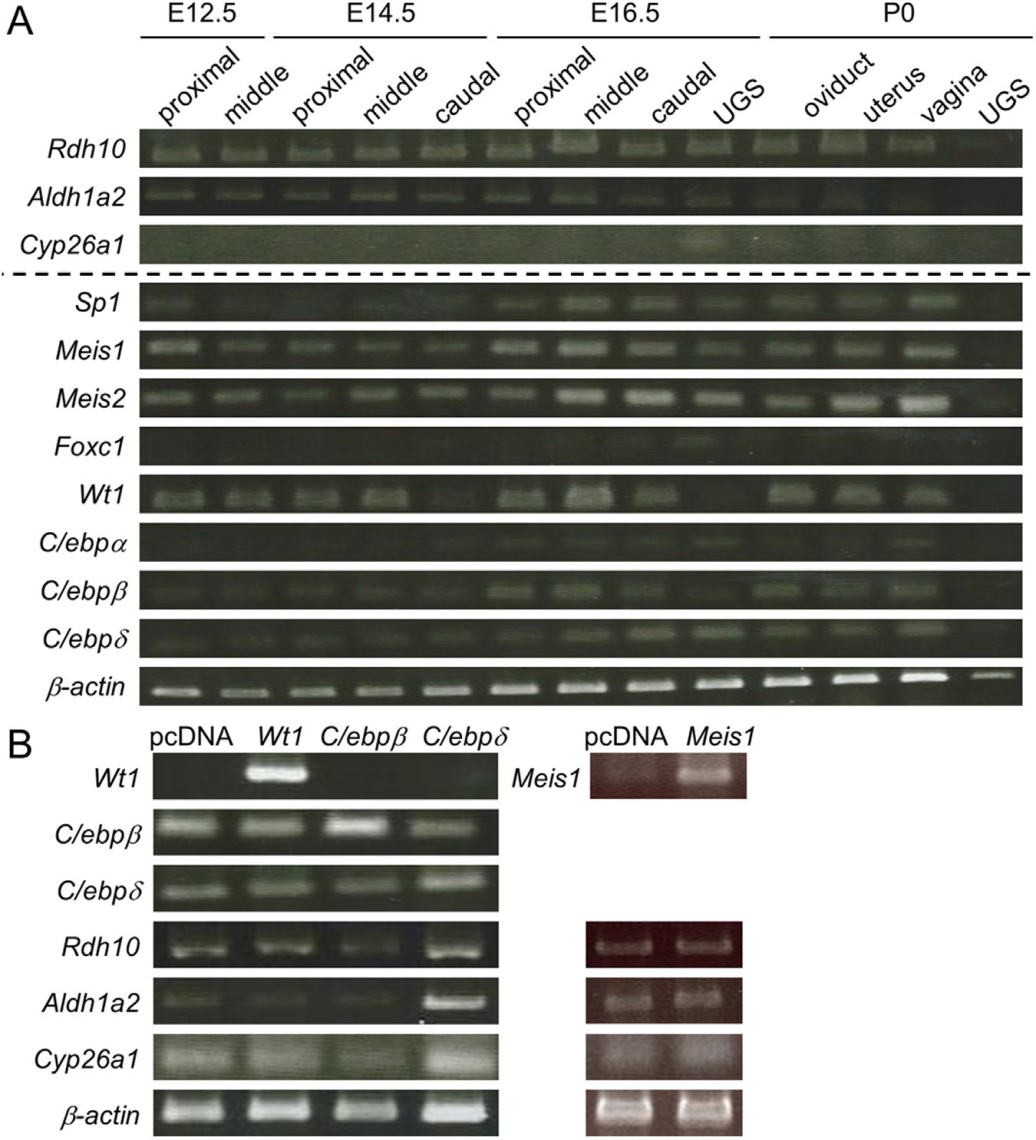
Ontogenic expression of *Rdh10*, *Aldh1a2* and *Cyp26a1*, *Sp1*, *Meis1*, *Meis2*, *Foxc1*, *Wt1*, *C/ebpα, C/ebpβ* and *C/ebpδ* in Müllerian ducts and UGS from E12.5 to P0 by RT-PCR (A). In P3US cells overexpressing *Wt1*, *C/ebpβ*, *C/ebpδ*, and *Meis1*, *Rdh10*, *Aldh1a2* and *Cyp26a1* expressions were analyzed by RT-PCR (B). (n = 3).

Since it is hypothesized that a borderline of retinoic acid signaling is determined by RA synthesis and metabolization in MDs, ontogenic expression of RA synthesis enzymes, *Rd10* and *Aldh1a2*, and RA metabolizing enzyme *Cyp26a1* was analyzed in MDs from E12.5 to P0. At E12.5, MDs have grown to the caudal region of the developing gonad [1]; therefore, the middle region of *meso*nephros (Fig. 1A) contained little MD cells. However, *Rdh10* and *Aldh1a2* expressions were already detected in the middle region of *meso*nephros before development of MDs (Fig. 2A). *Aldh1a2* expression was decreased at the caudal region from E12.5 to P0, and *Rdh10* expression level was similar in all samples except for UGS at P0. *Cyp26a1* was mainly expressed in UGS at the embryonic stages as mentioned above. Therefore, in MDs, RA signaling may be mainly controlled by the gradient of *Aldh1a2* expression and *Cyp26a1* expression at UGS.

Transcription factors stimulating *Aldh1a2* promoter activity are reportedly *Meis1*, *Meis2*, *C/ebpα*, *C/ebpβ*, *C/ebpδ*, and *Wt1* in epicardium, *Sp1* in dendritic cells and *Foxc1* in somite of zebrafish [19], [20], [21], [22]. Thus, ontogenic expression of the transcription factors was analyzed in MDs and UGS from E12.5 to P0 (Fig. 2A). *Meis1*, *Meis2*, *C/ebpβ*, *C/ebpδ* and *Wt1* were expressed in MDs from E12.5 to E16.5. Little *Wt1* expression was detected in caudal region at E14.5 and UGS at E16.5 and P0. *Sp1*, *Foxc1* and *C/ebpα* expressions were not detected in MDs at E12.5 and E14.5. These results suggest that transcription factors such as *Meis1*, *Meis2*, *C/ebpβ*, *C/ebpδ* and *Wt1* are candidates for activators of *Aldh1a2* promoter in MDs.

### 3.3 Effect of transcription factors on *Aldh1a2* expression in P3US cells

The effects of the transcription factors on *Aldh1a2* expression was analyzed in P3US cells derived from uterus mesenchyme at P3 with overexpression, since RA signaling is localized in MD mesenchyme [2]. Expression of *Wt1*, *C/ebpβ*, *C/ebpδ* and *Meis1* was confirmed in the overexpressing P3US cells (Fig. 2B). Overexpression of *Meis2* was unsuccessful although *Meis2*-overexpression vector was transfected in the cells. In *C/ebpδ*-overexpressing cells, *Aldh1a2* expression was strongly stimulated, and *Rdh10* expression and *Cyp26a1* expression were slightly stimulated. Unfortunately, knockdown of *C/ebpδ* in P3US cell line by retrovirus infection of *C/ebpδ*-sort hairpin RNA and lipofection of *C/ebpδ*-small interfering RNA was unsuccessful (data not shown).

**Fig. 3:**
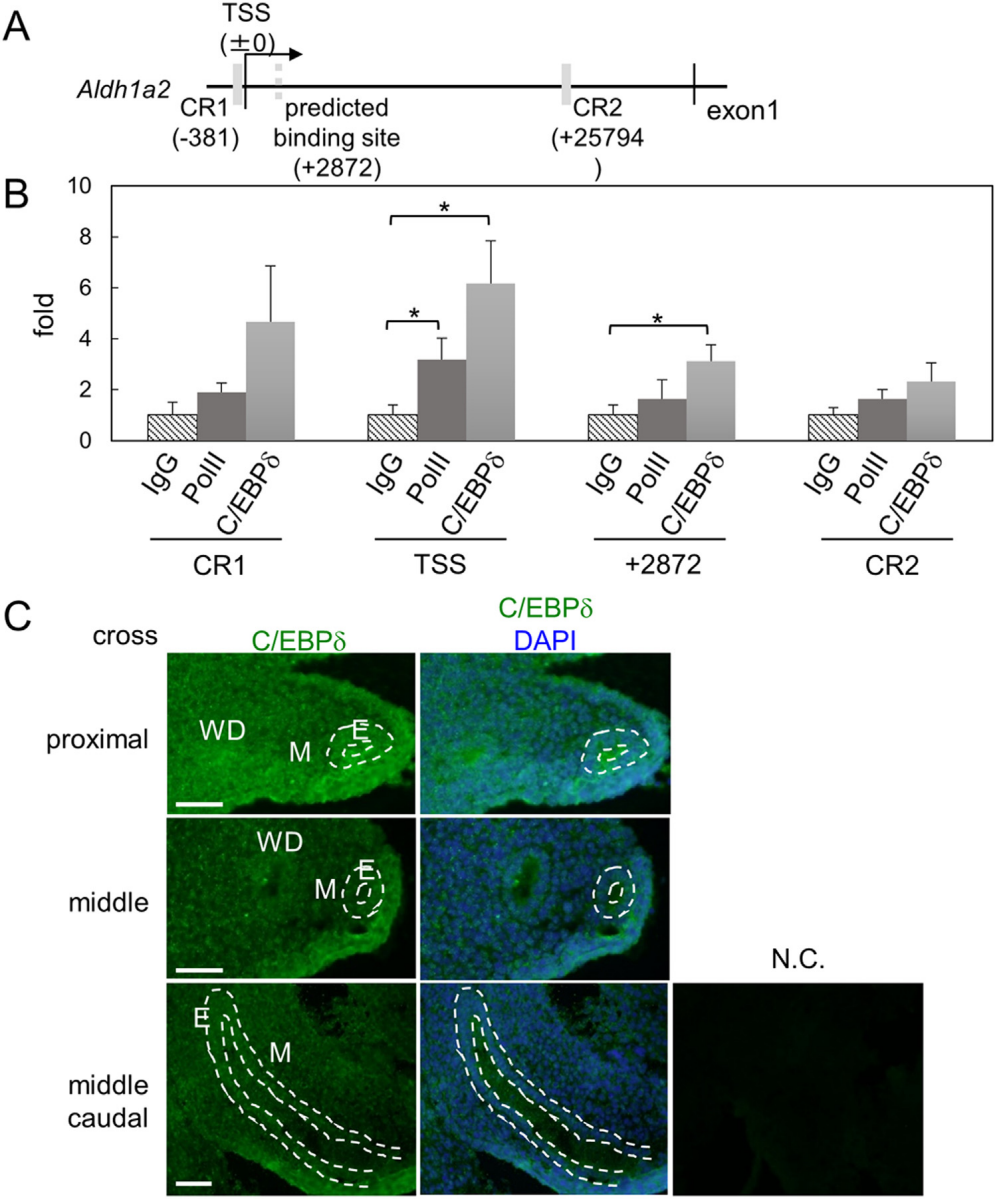
Predicted C/EBPδ binding sites exist in conserved region (CR1 and CR2; gray bold lines) of *Aldh1a2* promoter sequence and between CR1 and CR2 (gray dotted line) (A). ChIP assay of CR1, TSS, +2872 bp and CR2 in the *Aldh1a2* gene promoter in Müllerian duct of E14.5 mice using antibodies against RNA polymerase II and C/EBPδ (B). Data was expressed relative to immunoprecipitations using control normal IgG (=1.0), (n = 5). *: *p* ≤ 0.05. Expression of C/EBPδ in Müllerian ducts at E14.5 by immunohistochemistry. N.C.: negative control using normal IgG. Green: C/EBPδ positive cells. White line: borderline between the epithelium and mesenchyme. WD: Wolffian ducts. M: mesenchyme. E: epithelium. Scale bar: 50 µm. (n = 3).

Huang et al. reported that C/EBPδ binding sites were on conserved region of *Aldh1a2* promoter sequence [19], and we searched predicted C/EBPδ binding sites between conserved region 1 and 2 with score matrix for C/EBPδ [23] (Fig. 3A). To investigate C/EBPδ binding to *Aldh1a2* promoter sequence in vivo, ChIP assays were performed in MDs at E14.5 (Fig. 3B). RNA polymerase II (PolII) bound to *Aldh1a2* transcription start site (TSS). C/EBPδ bound to *Aldh1a2* TSS and the promoter region at + 2872 bp, indicating that C/EBPδ directly binds to *Aldh1a2* promoter in vivo.

Localization of C/EBPδ protein was investigated in cross sections of MDs at E14.5 by immunohistochemistry (Fig. 3C). C/EBPδ was expressed in the nuclei of MD epithelium and mesenchyme. Expression of C/EBPδ was higher in proximal region than that in caudal region. These results suggest that C/EBPδ is mainly localized in the nuclei of proximal MD mesenchyme.

### 3.4 Induction mechanism of gradient expression of protein in MD mesenchyme

**Fig. 4:**
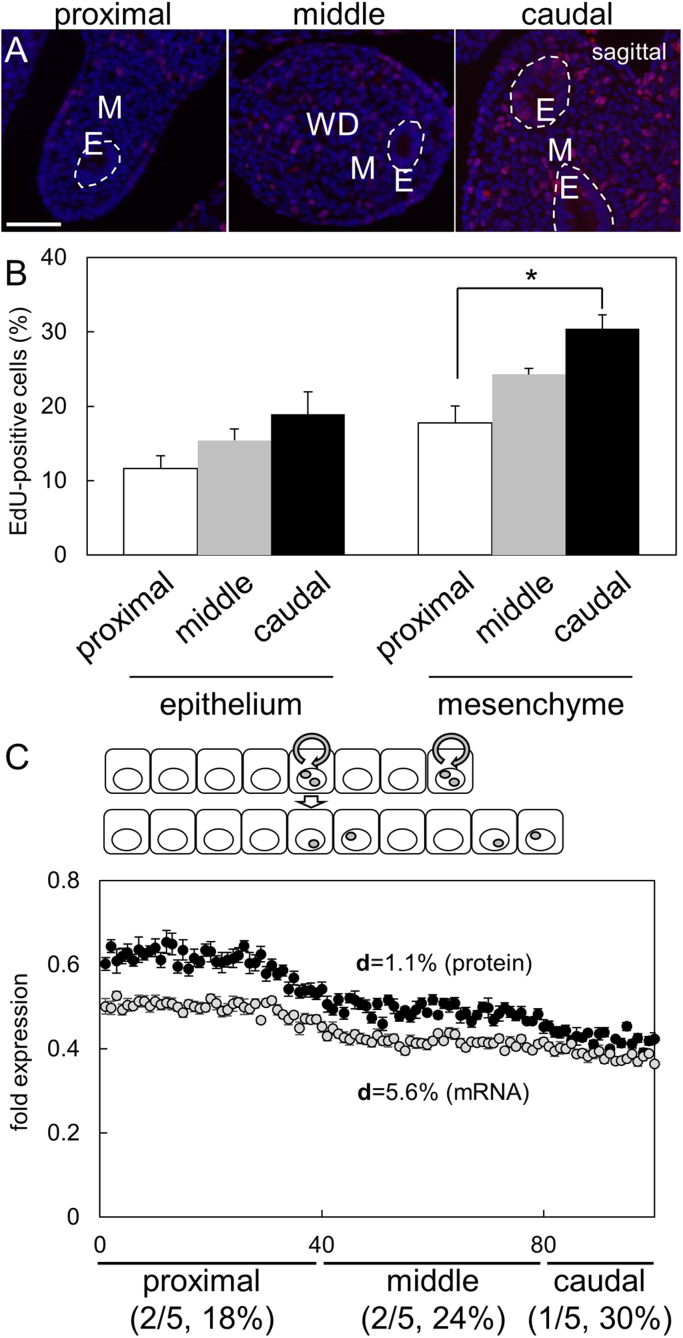
EdU staining in Müllerian ducts at E14.5 (n = 5–7). Red: EdU positive cells. White line: borderline between the epithelium and mesenchyme. WD: Wolffian ducts. M: mesenchyme. E: epithelium. Scale bar: 50 µm. The rate of EdU positive cells was presented (B). *: *p* ≤ 0.05. mRNA and protein expression levels were calculated on a theoretical model (C). d (degradation constant)= 5.6% (mRNA) or 1.1% (protein). Cell proliferation separated them at half quantity, and cell proliferation rate in proximal (2/5 head region in all cells), middle (2/5 following region) or caudal region (1/5 last region) was 18, 24 or 30%.

Although *C/ebpδ* mRNA expression was maintained at the same level in proximal, middle and caudal regions (Fig. 2A), C/EBPδ protein was mainly localized in the proximal region (Fig. 3C). Half-life of protein (median = 46 h) is longer than that the mRNA (median = 9 h) [24]; therefore, protein expression is reflect in various cell condition. Orvis and Behringer showed that MD epithelial cells highly proliferated at the tip of ducts [1]. If mitosis takes place in cells, the contents of proteins must be equally divided into daughter cells. Thus, we hypothesized that difference of mitosis frequency induces a gradient expression in MD mesenchyme. In fact, frequency of mitosis was significantly high in caudal mesenchyme compared with that in proximal mesenchyme at E14.5 by EdU staining (Fig. 4A, B). We then formularized a mathematical model of mRNA or protein level in MDs using parameter of frequency of mitosis (18, 24 or 30%/1 h in proximal, middle or caudal region) (Fig. 4C). When the value of **d** is 1.1%/1 h (predicted by half-life of protein), frequency of mitosis induces a gradient of protein level, but not the due to the value of **s**, or **d** = 5.6% (predicted by 9 h half-life of mRNA). These results suggested that constant synthesized and degraded protein represent gradient expression in MDs.

## 4 Discussion

The present study demonstrated that CYP26A1 was expressed not only in caudal MDs but also UGS connected with MDs, and in MD elongation, high proliferative activity at caudal region caused dilution of proteins involving in RA synthesis (e.g. ALDH1A2 and C/EBPδ). Point mutation of lipoma HMGIC fusion partner-like 2 gene leads failure of the connection to UGS and vaginal agenesis [25], supporting that the connection to UGS is essential for vaginal development. Therefore, MD morphogenesis (i.e. elongation and connection to UGS) generates a borderline of RA signaling causing fate determination of uterine and vaginal mesenchyme.

*Cyp26a1* mRNA was expressed in UGS, and CYP26A1 protein was expressed in all regions of MD mesenchyme and the epithelium of caudal MD and UGS. In contrast, the expression of ALDH1A2 and RA signaling was high in proximal and middle MDs [2], indicating that the distribution was opposite to CYP26A1. In early pregnancy, CYP26A1 blocking in uterus with an aberrant regulation of NK cells caused pregnancy failure [26]. In adult mice, *Cyp26a1* expression levels were 15.5 ± 1.7 at diestrus and 48.8 ± 12.9 folds at estrus compared with that in middle MD [2]. Semi-quantitative analysis of Fig. 1B showed that *Cyp26a1* expression level was 168.7 ± 66.9 folds in UGS compared with middle MD, suggesting that CYP26A1 level was sufficiently high for degradation of RA in UGS as well as in adult uterus. Thus, CYP26A1 was involved in the fate determination of vaginal mesenchyme. In caudal MD, ALDH1A2 protein is slightly detected in the mesenchyme [2]. In caudal MD and UGS, CYP26A1 protein was mainly expressed in the epithelium. In adult uterus, RA is synthesized in the stroma, whereas RA is metabolized in the epithelium [27]. Therefore, in caudal MD, RA is synthesized in the mesenchyme and metabolized in the epithelium.

C/EBPδ stimulated *Aldh1a2* expression, and it was directly bound to *Aldh1a2* promoter in vivo, indicating that C/EBPδ directly regulates *Aldh1a2* expression in MDs. However, the expression of *C/ebpδ* and *Aldh1a*2 showed reverse correlation in MD development (Fig. 2A), and female C/EBPδ knockout mice are viable and fertile [28], suggesting that C/EBPδ is one of the factors regulating *Aldh1a2* expression. *Aldh1a2* was already expressed in *meso*nephros at E7.5-E10.5 [29], and we also detected the expression at E12.5 before MD development. Furthermore, RA treatment to pluripotent cells induces differentiation into *Osr1*-expressing cells [30], [31], [32], which are mesonephric cells in mesoderm [33]. Collectively, RA synthesizing system including transcription factors regulating *Aldh1a2* expression is essential for mesonephric development; therefore, in MDs, the system may be diverted to the mesenchymal differentiation.

In human, some disorders are reported for atresia and agenesis of vagina and cervix [34], [35]. Although human vaginal development also include steps of MD elongation and connection of MD to UGS [36], vaginal epithelial origin is different between human in which vaginal epithelium is derived from UGS [36] and mouse in which it is derived MD [3]. Furthermore, in patient of Mayer-Rokitansky-Küster-Hauser syndrome, one of the major vaginal abnormality, the epithelium of closed vaginal antral mucosa are expanded to the entire vagina by surgery and treatment of fibroblast growth factor and differentiates into vagina-like estrogen receptor-expressed stratified epithelium [37]. Taken together, epithelial stratification of MD is necessary only for cervical development in human. Consequently, disorders of cervical abnormality with vaginal presence may be caused by elongation of MD or connection to UGS at development.
