# Supplementary material for: Elongation of Müllerian ducts and connection to urogenital sinus determine the borderline of uterine and vaginal development
Source: Biochem Biophys Rep. 2018 Nov 30;17:44–50. doi: 10.1016/j.bbrep.2018.10.013 (PMC6279966; doi:10.1016/j.bbrep.2018.10.013)
Supplement: Supplementary file 2 — Supplementary material [file mmc2.docx]

Supplemental Table Primer for RT-PCR, overexpression and ChIP

| Gene | Forward sequence (5'→3') | Reverse sequence (3'→5') |
| --- | --- | --- |
| *Cyp26a1* | AAGGCGCGGAACCTTATACA | TCTTGGCGCGAATGTTCTC |
| *Cyp26b1* | TCCACCTTCTATCGGCAATCT | CAGTTGGATCTTGGGCAGGT |
| *Aldh1a2* | TGCAGGCTGGGCTGATAAAA | ACAGCGTAGTCCAAGTCAGC |
| *Rdh10* | CTGCAAGCTGGAAAAGGTGAG | ACACGACGGCTTCAAAAGGA |
| *β-actin* | TGTTACCAACTGGGACGACA | TCTCAGCTGTGGTGGTGAAG |
| *Meis1* | CGTGGCTGTTCCAGCATCTA | CATTGGCTGTCCATCAGGGT |
| *Meis2* | CCATGCTTCCCAGAGTGGAG | GGGTGTGTGAGATGCTGGAA |
| *Sp1* | GACTCGTCGGGAAGCATGTA | AGTGTGCTCGGAGATGTGAG |
| *C/ebpα* | TTCGGGTCGCTGGATCTCTA | CTCATCTTAGACGCACCGAGT |
| *C/ebpβ* | CAAGCTGAGCGACGAGTACA | CTGCTCCACCTTCTTCTGC |
| *C/ebpd* | ACTCCTGCCATGTACGACGA | GAAGAGGTCGGCGAAGAGTT |
| *Wt1* | TCCGAGGCATTCAGGATGTG | ATGAGTCCTGGTGTGGGTCT |
| *Foxc1* | AAGGACGCAGTGAAGGACAA | CGTACCGTTCTCCGTCTTGAT |
| *Meis1* (ORF) | GGGGATCCATGGCGCGGTACGACGACCTAC | GGGCTCGAGCTACTGTGAATGTCCATGACT |
| *Meis2* (ORF) | GGATATCATGGCGCAAAGGTACGATGAGCTGC | GGGCTCGAGCTATTGGGCATGAATGTCCATAACC |
| *C/ebpβ* (ORF) | GGGGATCCATGGAAGTGGCCAACTTCTACTACG | GGGCTCGAGCTAGCAGTGGCCCGCCGAGGCCAGC |
| *C/ebpδ* (ORF) | GGGGATCCATGAGCGCCGCGCTTTTCAGCCTGG | GGGGATATCTTACCGGCAGTCGGCGCCGGTGGGC |
| *Wt1* (ORF) | GGGAATTCCTGGACTTCCTGTCGCAGGAGC | GGGCTCGAGTCAAAGAGCTGGAGTTTGGTC |
| *Aldh1a2* TSS | GCCGTATGCAAATGTCCTCCTG | CGCTCGCTGTATATAGGCAGGT |
| *Aldh1a2* BS | AGACTGCTCAATAGCTGGTCTG | GTGTATGCTCCCACAACACG |
| *Aldh1a2* CR1 | ATGCAAATGTCCTCCTGGGC | TATAGGCAGGTGTCAAGCGG |
| *Aldh1a2* CR2 | GAACTCTCTGGGTGTTGTCCA | AGTGACCATTTAGTGGCCTAGC |
